# An outbreak of *Cryptosporidium parvum* linked to pasteurised milk from a vending machine in England: a descriptive study, March 2021

**DOI:** 10.1017/S0950268822001613

**Published:** 2022-10-28

**Authors:** Anya Gopfert, Rachel M. Chalmers, Sarah Whittingham, Laura Wilson, Maria van Hove, Claire F. Ferraro, Guy Robinson, Nick Young, Bayad Nozad

**Affiliations:** 1 Health Protection Team, UK Health Security Agency South West, Bristol, UK; 2 Cryptosporidium Reference Unit, Public Health Wales Microbiology and Health Protection, Singleton Hospital, Swansea SA2 8AQ, UK; 3 Swansea University Medical School, Singleton Park, Swansea SA2 8QA, UK; 4 Local Authority*, South West (*local authority name withheld due to confidentiality); 5 Animal & Plant Health Agency, Starcross Veterinary Investigation Centre, Exeter, Devon EX8 1BA, UK

**Keywords:** Bacterial infections, hygiene – food, outbreaks, public health

## Abstract

We describe the investigations and management of a *Cryptosporidium parvum* outbreak of linked to consumption of pasteurised milk from a vending machine. Multiple locus variable number of tandem repeats analysis was newly used, confirming that *C. parvum* detected in human cases was indistinguishable from that in a calf on the farm. This strengthened the evidence for milk from an on-farm vending machine as the source of the outbreak because of post-pasteurisation contamination. Bacteriological indicators of post-pasteurisation contamination persisted after the initial hygiene improvement notice. We propose that on-farm milk vending machines may represent an emerging public health risk.

## Background

*Cryptosporidium* is a genus of protozoan parasites which causes the gastrointestinal disease cryptosporidiosis, a mandated notifiable disease in the UK in humans. Although many species of *Cryptosporidium* exist most human infections are caused by *C. hominis*, which is host adapted to humans, and *C. parvum* which can additionally infect a range of hosts including domestic livestock. *Cryptosporidium* infects the small intestine causing watery diarrhoea, the main symptom of human infection. Diarrhoea usually lasts up to two weeks, with the potential for recurrence. While most people suffer mild to moderate diarrhoea, cryptosporidiosis can cause complications and be life-threatening for immunocompromised individuals [1].

Over 4000 cases of cryptosporidiosis are reported in England and Wales annually; in 2017, South West England had the highest rate of cryptosporidiosis with 10.6 cases per 100 000 population [2]. *Cryptosporidium* outbreaks are most often associated with swimming pools and visits to petting or open farms [3]. Few foodborne outbreaks of *Cryptosporidium* are reported; however, this is potentially due to under ascertainment and underreporting [2, 4]. Cryptosporidiosis outbreaks have been reported due to consumption of unpasteurised milk [5].

We describe the epidemiological, microbiological, environmental investigation and response to an outbreak of *C. parvum* linked to consumption of pasteurised milk from a vending machine in a rural area of South West England- an increasingly common method for farmers to sell their produce [6].

## Methods

### Outbreak detection

In March 2021, the health protection team were notified by an Environmental Health Officer (EHO) from the local authority of three cases of laboratory-confirmed symptomatic *Cryptosporidium* infection with a common exposure to a farm-based milk-vending machine. Although onset dates were spread over 35 days, the common exposure was notable and of public health concern. The day of notification for this outbreak is described as day 0.

After notification, an Outbreak Control Team (OCT) was established. The OCT included representatives from the health protection team, local authority Environmental Health department, the Animal and Plant Health Agency (APHA), Field Services (field epidemiology team; FS), the Food Standards Agency (FSA) and the national Cryptosporidium Reference Unit.

### Epidemiological

Case finding was undertaken. All persons with a laboratory-confirmed *Cryptosporidium* infection in a district of South West England between day −130 and day +20 recorded on a national health protection case management system (HPZone) were reviewed. This time period included at least two incubation periods before the first case, and at least two incubation periods after a hygiene improvement notice was served (day −30). For all cases, questionnaires – routinely administered for notified cases – were reviewed for milk consumption and/or any milk obtained from a milk vending machine or a farm. If no questionnaire was available cases were contacted to request completion; three attempts were made to contact cases. A copy of the questionnaire can be found on the UK Government website [7]. Additionally, data were reviewed by the local authority to look for any increase in *Cryptosporidium* cases.

Further case finding was undertaken by the national Cryptosporidium Reference Unit. All available samples from cases of *C. parvum* in the affected district between day −130 and day +20 were subtyped as described in the following section.

The following case definitions were used:

- Probable case: a laboratory confirmed case of *Cryptosporidium*, with reported exposure to milk from a vending machine at the farm in question, without molecular microbiology.
- Confirmed case: a laboratory confirmed case of *Cryptosporidium* with reported exposure to milk from a vending machine at the farm in question with molecular microbiological evidence.

### Microbiological

In the absence of a standard accredited method to test milk for *Cryptosporidium,* faecal sampling of animals on the farm was undertaken by a Veterinary Investigation Officer from APHA. Twenty-eight samples were taken, with calves targeted as animals less than six weeks of age are most likely to be affected. Faeces were tested for *Cryptosporidium* oocysts using immunofluorescence microscopy (Cellabs, TCS BioScience) by the APHA parasitology laboratory, Carmarthen.

At the national Cryptosporidium Reference Unit, to identify species, DNA was extracted from *Cryptosporidium*-positive stool samples (human and calf) and subjected to a duplex PCR targeting parts of the *C. hominis* A135 and *C. parvum* Lib13 genes [8].

The *C. parvum* samples were then subtyped by sequencing part of the hypervariable gp60 gene [3]. To further discriminate subtypes, a seven-locus multiple locus variable number of tandem repeats analysis (MLVA) was undertaken by fragment sizing amplicons generated in three-plex and four-plex assays [9].

### Environmental

The farm was visited by the EHOs, APHA and the FSA. The farm is a multi-generational family run dairy farm where the majority of milk is sold for wholesale processing. A small quantity of milk is pasteurised on-site to enable diversification of the farm, and cheese and eggs are also sold.

The visits included inspection of the environment for the herd and for milk pasteurisation. EHOs undertook multiple visits to the farm to complete an initial hygiene inspection, collect samples and conduct revisits to assess compliance with the hygiene improvement notices. Usually, samples of milk would be tested for the suspected pathogen, but it is not possible to test for Cryptosporidium in milk therefore sampling focussed on swabbing of pasteurisation equipment and the vending machine, and samples of milk were taken from the pasteuriser and the vending machine to test for a range of organisms, including Enterobacteriaceae. The APHA visited the farm, took samples from the animals and supported the farmer. The FSA also conducted a thorough inspection to identify causes of cross-contamination. Because any farm environment will have a number of pathogens, this investigation aimed to prevent further human cases and therefore preventing cross-contamination of the vending machine was the main focus of the investigation.

## Results

### Epidemiological

**Fig. 1.**
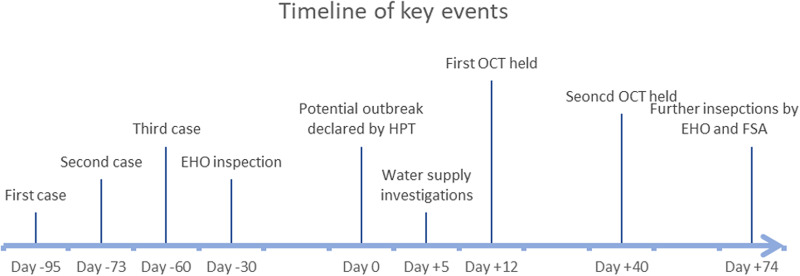
Timeline of key events.

Figure 1 summarises key events in the outbreak investigation. Between day −130 and +20, 33 cases of cryptosporidiosis were identified in the South West region. Questionnaire data were available for 26/33 (79%). No alternative common exposures were identified, and no further cases beyond the initial three (two confirmed and one probable as per our case definition) had consumed milk from a milk-vending machine.

The details of three cases confirmed to have consumed pasteurised milk purchased from the milk-vending machine are summarised in Table 1. The cases included two confirmed cases (male (51 years), female (78 years)) and one probable case (male (41 years)).

**Table 1.** Details of cases associated with the outbreak

Case no.	Sex, and age at time of diagnosis	Symptom onset date	Test date	Sample sent to reference lab?	Case definition
1	Male, 41 years	Day −95	Day −70	No	Probable
2	Male, 51 years	Day −73	Day −62	Yes	Confirmed
3	Female, 78 years	Day −60	Day −43	Yes	Confirmed

No evidence of a statistically significant increase of cryptosporidiosis in the district was found in surveillance data.

### Microbiological

For case-finding, the reference laboratory identified five samples in the relevant time-period and district. This included two of the three cases associated with the milk vending machine (the third sample was not sent to the reference laboratory). The two cases associated with the milk vending machine were the same *C. parvum* gp60 subtype (IIaA19G1R1). The other three cases identified as part of the case finding search were different subtypes, had no evidence of exposure to the farm or the milk-vending machine, and were therefore not associated with this outbreak.

From the sampling of farm animals, one sample from a calf was positive for *Cryptosporidium* and sent for subtyping at the reference laboratory. It was the same gp60 subtype (IIaA19G1R1) as the human samples.

To further investigate relatedness of the two human samples and calf sample, the reference laboratory undertook MLVA. This method had been validated but was not yet accredited and was being trialled for application to outbreaks in real time [9]. Investigation of the fragment sizes indicating allele variation showed that *C. parvum* from the two human cases and one calf sample were indistinguishable at the seven loci investigated, with a MLVA profile of 4-14-5-7-18-25-17 [9].

EHOs took further samples from the milk vending machine for hygiene indicators, two months after the initial hygiene improvement notice. These samples had unsatisfactory levels of Enterobacteriaceae, an indicator of post-pasteurisation contamination [10].

### Environmental

The EHO inspected the farm and rated it as ‘major improvement necessary’ and served hygiene improvement notices, as illustrated in Figure 1. Although pasteurisation was deemed to be achieved and validated with phosphatase test results and temperature records, specific concern was noted regarding the cleaning of equipment and the vending machine churns, particularly after pasteurisation had occurred. EHOs continued to work with the food business operator throughout the outbreak investigation to implement required improvements.

The FSA inspected the farm after ongoing unsatisfactory levels of Enterobacteriaceae were identified in milk samples concluding that inadequate cleaning after pasteurisation was a potential contamination route and made recommendations for improvements.

## Discussion

This study describes three cases of *C. parvum* infection following consumption of pasteurised milk from an on-site farm vending machine. Descriptive epidemiology, environmental inspections and advanced microbiological evidence was utilised to link cases, milk consumption and the dairy herd, suggesting post-pasteurisation contamination as the source of outbreak; further supported by the presence of Enterobacteriaceae in environmental samples from the vending machine.

To our knowledge this is the first documented *Cryptosporidium* outbreak associated with pasteurised milk from a milk-vending machine. This investigation employed MLVA of *C. parvum*, a new application of this method, which strengthened the evidence and may be suitable for future outbreak investigations [9]. The use of MLVA in this study provided robust evidence linking the genotype found in the herd (via the calves) to those obtained from cases. Although the validation of the assay had included testing samples from historical outbreaks, this was the first time the MLVA scheme had been applied during an ongoing investigation [9]. There is good evidence from the validation data that isolates with same MLVA profile are likely to be epidemiologically linked. Among the 136 epidemiologically unrelated samples in the validation panel, the majority of MLGs (81/102, 79%) were unique. The MLVA profile identified in this outbreak has not been seen previously at the reference unit.

Outbreaks of human cryptosporidiosis associated with unpasteurised milk have been documented in the UK, USA and Australia [6]. *C. parvum* is a common cause of enteritis in calves, and oocysts can survive in the environment leading to contamination of premises and utensils. Routine cleansing and disinfection procedures on-farm are not sufficient to remove oocysts. General on-farm hygiene, pasteurisation and post-pasteurisation hygiene are critical control points.

Guidance and training on Hazard Analysis and Critical Control Point principles, and implementation of good manufacturing processes are essential for any food business to operate safely. Human factors have been found responsible for outbreaks of food-borne pathogens associated with on-farm pasteurisation previously [11, 12]. Small-scale ‘traditional’ pasteurisation processes are associated with a higher risk of microbiological contamination compared to commercial dairies [13, 14]. Post-pasteurisation contamination is a common source of (re)introduction of foodborne pathogens [15], however we found no specific reports of *Cryptosporidium* in the literature associated with pasteurised milk.

There has been an increase in the direct sale of milk products across South West England in recent years [6]. Farmers are sometimes unaware they need to register as Food Business Operators (FBOs) to sell direct-to -consumer products, therefore no precise estimates of the number of FBOs selling on-farm pasteurised milk exist. A recent survey of 13 lower-tier local authorities estimated a 63 on-farm pasteurisers and 104 milk-vending machines in the South West [6]. Potential risks to public health identified included difficulties with effective cleaning of equipment, safe transportation of pasteurised milk on-farm, failure of pasteurisation processes, risk of post-pasteurisation contamination from the environment and a lack of guidance for FBOs and EHOs about milk vending machines.

This outbreak demonstrated the importance of multi-agency stakeholders managing an outbreak of *C. parvum* associated with a milk vending machine and the importance of case questionnaires that include relevant exposure/food and beverage consumption questions. This outbreak also demonstrated applications for MLVA microbiological analysis, and its role in strengthening evidence for outbreak investigations. It also provides evidence for the value of sampling cattle for Cryptosporidium by enabling in-depth characterisation of isolates in a one-health approach.

To tackle the emerging public health risk of post-pasteurisation contamination associated with on-site milk vending machines, EHOs must continue to proactively identify and regulate on-farm pasteurisers and milk vending machines according to food business registration law. It is recommended that existing on-farm pasteurisation guidance is reviewed [10], to include milk vending machines. This may include resources for EHOs (inspection checklists, training) and the provision of information and training to manufacturers, users and suppliers of equipment.

## Data Availability

This manuscript describes a limited series of three cases in detail, and as such there is no broader dataset for release. Data regarding individual cases are held on a case management system and sharing of this confidential data would not be appropriate. Any queries regarding the data can be directed to the corresponding author.
